# Topoisomerase IIβ Binding Protein 1 c.*229C>T (rs115160714) Gene Polymorphism and Endometrial Cancer Risk

**DOI:** 10.1007/s12253-013-9737-7

**Published:** 2013-12-18

**Authors:** Ewa Forma, Katarzyna Wójcik-Krowiranda, Paweł Jóźwiak, Agnieszka Szymczyk, Andrzej Bieńkiewicz, Magdalena Bryś, Anna Krześlak

**Affiliations:** 1 Department of Cytobiochemistry, University of Łódź, Pomorska 141/143, 90-236 Łódź, Poland; 2 Clinical Division of Gynecological Oncology, Medical University of Łódź, Pabianicka 62, 93-509 Łódź, Poland

**Keywords:** Topoisomerase IIβ binding protein 1, Polymorphism, Genetic variation, Endometrial cancer

## Abstract

TopBP1 (topoisomerase IIβ binding protein 1) protein is involved in DNA replication, DNA damage checkpoint response and transcriptional regulation. In this study we investigated whether alterations in the TopBP1 gene can influence the risk of endometrial cancer. We examined the association between five single nucleotide polymorphisms (rs185903567, rs116645643, rs115160714, rs116195487, and rs112843513) located in the 3′UTR region of the TopBP1 gene and endometrial cancer risk as well as allele-specific gene expression. One hundred twenty-one endometrial cancer patients were genotyped for these SNPs. Allele-specific TopBP1 mRNA and protein expressions were determined by real time PCR and western blotting methods, respectively. Only one SNP (rs115160714) showed an association with endometrial cancer. Compared to homozygous common allele carriers, heterozygous for the T variant had significantly increased risk of endometrial cancer [adjusted odds ratio (OR) = 5.59, 95 % confidence interval (CI): 1.96–15.91, *p* = 0.0003]. Mean TopBP1 mRNA and protein expression were higher in the individuals with the CT genotype. There was a significant association between the rs115160714 and tumor grade and FIGO classification. Most carriers of minor allele had a high grade tumors (G3) classified as FIGO III/IV. The results of our study raise a possibility that a genetic variation of TopBP1 may be implicated in the etiology of endometrial cancer.

## Introduction

Endometrial cancer is the most common malignancy of the female reproductive tract. It accounts each year for approximately 142.000 new cases diagnosed worldwide, and for 42.000 deaths. Endometrial cancer is the seventh most common malignant disorder and its incidence is expected to increase in the near future due to the increase in life span expectancy and obesity [1]. However, despite great progress in the endometrial cancer studies, the molecular mechanisms that contribute to endometrium carcinogenesis remain poorly understood. Thus, there is a necessity to identify all endometrial cancer susceptibility genes.

The latest international prospective cohort study showed that risk of endometrial cancer is higher in BRCA1 mutation carriers than in the general population [2]. TopBP1 (topoisomerase IIβ binding protein 1) protein displays structural and functional similarities with BRCA1 and is involved in DNA replication, DNA damage checkpoint response and transcriptional regulation. TopBP1 gene comprising 28 exons in located on chromosome 3q22.1 and encodes a 1522 amino acid proteins [3]. The most characteristic feature of TopBP1 is that it has eight BRCT (BRCA1 C-terminal) domains [4, 5]. These domains are involved in interaction with other proteins as well as in interaction with single and double-stranded DNA [6]. The C-terminal region of TopBP1 containing two BRCTs is responsible for interaction with topoisomerase. Following ionizing radiation, TopBP1 is recruited to DNA breaks and co-localizes with Nbs1 (Nijmegen breakage syndrome 1), BRCA1 and 53BP1 (p53-binding protein 1) in nuclear foci. TopBP1 and BRCA1 also co-localize with proliferating cell nuclear antigen (PCNA) at stalled replication forks after a replication block [3]. TopBP1 has been established as an essential activator of ATR (ATM and RAD3-related) kinase. ATR plays a crucial role in maintenance of genomic integrity by delaying cell division in the presence of DNA damage or replication stress [7].

The results of our earlier studies showing association between TopBP1 polymorphism and breast cancer risk prompted us to investigate whether such genetic alterations can influence the endometrial cancer risk. In present study we tested the effect of five SNPs [rs185903567 (G/A), rs116645643 (A/G), rs115160714 (C/T), rs116195487 (C/G), and rs112843513 (C/delC)] in the 3′UTR (3′untranslated region) of TopBP1 gene on endometrial cancer risk as well as on allele-specific mRNA/protein expression. We correlated obtained results with clinicopathological characteristics.

## Materials and Methods

### Study Population

This study involved 121 women with endometrial carcinoma (age range 31–84, mean age 63.84 ± 10.24) recruited between March 2009 and December 2012. The patients had a confirmed diagnosis of endometrial carcinoma based on histopathological evaluation and were under treatment at the Clinical Division of Gynecological Oncology, Medical University of Łódź. None of the recruited patients received preoperative chemo- or radiotherapy. Patients diagnosed with previous endometrial tumors or with tumors located elsewhere were excluded. The clinical stage of the disease was defined according to the FIGO criteria. Histological grade was based on the degree of glandular differentiation, and tumors were graded as: G1 (percentage of solid growth in the tumor mass up to 5 %); G2 (percentage of solid growth between 6 and 50 %); G3 (percentage of solid growth above 50 %). The depth of myometrial invasion was defined as the percentage of the myometrium invaded by the tumor. The distributions of sociodemographic characteristics and clinical characteristics of the patients are shown in Table 1.

**Table 1:** Selected baseline characteristics of endometrial cancer cases

	Cases (n, %) (*n* = 121)
Age	63.84 ± 10.23
Grade	
1	22 (18.2)
2	80 (66.1)
3	19 (15.7)
FIGO	
I	77 (63.6)
II	19 (15.7)
III/IV	25 (20.7)
Myometrial invasion	
Inner half	57 (47.1)
Outer half	53 (43.8)
n/a	11 (9.1)
Lymph node metastasis	
Negative	97 (80.1)
Positive	24 (19.9)

As a control in the study of polymorphisms we used the results obtained and published earlier for a group of 556 healthy Polish individuals [8]. They were collected from the hospital routine controls of health and used as control. They were non-related women, that have never been diagnosed with endometrial tumors, other tumors or chronic disease and were randomly selected and frequency matched to the cases on age (age range 34–83, mean age 51.27 ± 11.18).

Informed consent was obtained from patients and controls, and the Ethical Committee approved the study.

### DNA Extraction and Genotyping

Each genomic DNA sample was extracted from endometrial cancer tissues and peripheral blood samples using FlexiGene® DNA Kit (Qiagen GmbH, Hilden, Germany). DNA concentration was determined by spectrophotometry using the Helios Alpha UV–Vis spectrophotometer system (Thermo Fisher Scientific Inc.). The absorbances at 230, 260 and 280 nm were measured on 2 μl of each sample. Concentration of DNA was estimated by spectrophotometric quantification at 260 nm. The overall purity was assessed by calculating the absorbance ratios 260/280 and 260/230. High values for both ratio (260/280 > 1.8, 260/230 > 2) are commonly accepted as good indicators for pure DNA. The single nucleotide polymorphisms (SNPs) rs185903567 (G/A), rs116645643 (A/G), rs115160714 (C/T), rs116195487 (C/G), rs112843513 (C/delC) located at the 3′UTR of TopBP1 gene were evaluated (http://www.ensembl.org/Homo_sapiens/Gene/Sequence?db=core;g=ENSG00000163781;*r*=3:133317019-133380737). These polymorphisms were analyzed by ASO-PCR (rs185903567, rs116645643, rs116195487) and RFLP-PCR (rs115160714, rs112843513).

### ASO-PCR Assays

Mutated or wild-type sequences were specifically amplified in a noncompetitive PCR reaction performed on DNA in 50 μL reaction mixture and PCR conditions as described below, using allele-specific and reverse primers as follows: for the rs185903567 SNP, F wildtype: 5′-TGAAGAATTCTGCTTCAGTA-3′ or F mutant: 5′-TGAAGAATTCTGCTTCAGTG-3′ and R: 5′- TTACAATTTCAGGTGTTCAAA-3′ (annealing at 57 °C; 64-bp PCR product); for the rs116645643 SNP, F wildtype: 5′-AAAGTTACCTGAAATAACAACTA-3′ or F mutant: 5′-AAAGTTACCTGAAATAACAACTG-3′ and R: 5′- AATGTGGTTTAACAGCAAGC-3′ (annealing at 55 °C; 61-bp PCR fragment); for the rs116195487 SNP, F wildtype: 5′-CTTGCTGTTAAACCACATTGAAGAG-3′ or F mutant: 5′-CTTGCTGTTAAACCACATTGAAGAC-3′ and R: 5′-TCATTAAACCTTGTGCTCAG-3′ (annealing at 58.5 °C; 234-bp PCR fragment). The sensitivity of this assay was determined for each mutation by amplification of 10-fold limited dilutions of 100 ng patient’s DNA at time of resistance in 100 ng healthy control DNA.

### RFLP-PCR Assay

PCR reactions were performed, using Perkin-Elmer DNA Thermal cycler 480, in a total volume of 50 μl to amplify TopBP1 rs115160714, rs112843513. The reaction mixtures consisted of 100 ng of genomic DNA and the following set of primers: 10 μM of rs115160714 primers (5′-CCCTTCTTGAGTTTTGAACACC-3′ and 5′-AAAGCAAAATCCATTACCTTGC-3′), rs112843513 primers (5′-GCCTGAGCACAAGGTTTAATG-3′ and 5′-ACAGATGCCAGGGTGCTC-3′). The DNA samples were amplified in the presence of 200 μmol dNTPs, 10 % dimethylsulfoxide, 1 x Taq polymerase buffer, 1.5 mM MgCl_2_ and 0.5U AmpliTaq Gold (PE Applied Biosystems, Foster, CA). The PCR condition for rs115160714 and rs112843513 comprised an initiation denaturation step at 94 °C for 4 min, followed by 30 cycles of 96 °C for 1 min, 59 °C for 1 min, 72 °C for 1 min and final extension step at 72 °C for 10 min. Subsequently, RFLP analysis was performed on 20 microliters each of the respective PCR products by subjecting them to the following restriction enzymes: at a 5U concentration: *BseR*I (at 37 °C for 3 h) for rs115160714 and *Ras*I (at 37 °C for 16 h) for rs112843513 (both from New England Biolabs, UK).

In the case of rs115160714 the lengths of fragments obtained by digestion of each 211-bp fragment by *BseR*I were 84 bp and 127 bp for wild type, and 211 bp for mutant type. For rs112843513, the lengths of fragments obtained by digestion of each 211-bp fragment by *Ras*I were 71 bp and 140 bp for wild type, and 211 bp for mutant type.

The products were analyzed by electrophoresis on 3 % agarose gels and ethidium bromide-stained. Positive and negative controls were included in each gel. Quality control was ensured by including a random 5 % of the samples as duplicates.

### Total RNA Extraction and cDNA Synthesis

Total RNA was extracted from endometrial cancer tissues using TRI Reagent® (Sigma Aldrich Corp. St. Louis, MO, USA) according to manufacturer’s protocol. RNA was eluted in 20 μl RNase-free water, quantified by spectrophotometry at 260 nm and stored at -20 °C. RNA with a 260/280 nm ratio in range 1.8–2.0 was considered high quality. First-strand cDNAs were obtained by reverse transcription of 1 μg of total RNA using RevertAid^TM^ First Strand cDNA Synthesis Kit (Fermentas UAB, Vilnius, Lithuania) following the manufacturer’s protocol.

### Real Time Quantitative PCR

For real-time PCR analysis of TopBP1 mRNA in pathological tissues, TaqMan® Gene Expression Assays (Applied Biosystems, Bedford, MA, USA) were used according to the manufacturer’s instruction. Before starting the real-time PCR analysis we used the NormFinder algorithm to select the best reference gene (http://www.mdl.dk). We chose GAPDH (glyceraldehyde 3-phosphate dehydrogenase) gene because it had the lowest stability value - 0.017. The fluorogenic, FAM labeled probes and the sequence specific primers for TopBP1 and GAPDH were obtained as inventoried assays Hs00199775_m1 and Hs99999905_m1, respectively (Applied Biosystems, Bedford, MA, USA). The reactions were performed in duplicate. A positive result was defined by a threshold cycle (Ct) value lower than 40 (the Ct value is determined by the number of cycles needed to exceed the background signal). Ct value of all positive results were lower than 30. Abundance of TopBP1 mRNA in studied material was quantified by the ΔCt method. ΔCt (Ct_*TopBP1*_-Ct_*GAPDH*_) values were recalculated into relative copy number values (number of copies of TopBP1 mRNA per 1000 copies of GAPDH mRNA).

### Western Blotting Analysis

Tissue homogenate was obtained from each sample in the presence of the serine protease inhibitor PMSF (phenylmethylsulfonyl fluoride) and 10 mM sodium molybdate. The protein content was estimated by modified Lowry method using bovine serum albumin as standard. Homogenate proteins (50 μg protein/lane) were resolved by 8 % SDS-PAGE and electroblotted onto Immobilon-P transfer membranes (Millipore, Bedford, MA, USA). The blots were incubated 1 h with rabbit polyclonal anti-TopBP1 (Abcam, Cambridge, UK) in a 1:1000 dilution. After being washed three times with TBST (Tris buffered saline with Tween-20), the membranes were incubated 1 h with goat anti-rabbit antibodies conjugated with horseradish peroxidase (1:5000 dilution). The membranes were again washed three times with TBST and incubated with peroxidase substrate solution (3,3′-diaminobenzidine -DAB). Gel-Pro® Analyzer software (Media Cybernetics Inc., Bethesda, MD, USA) was used for densitometry analysis of protein bands. The integrated optical density (IOD) of the bands, in a digitized picture, was measured.

### Quality Control

For quality control purposes, 10 % of samples were randomly selected, and sequence analysis performed, with 100 % concordance to the genotype. Laboratory personnel were unable to distinguish among case, control, and quality control samples.

### Statistical Data Analysis

Genotype distributions were evaluated for agreement with Hardy–Weinberg equilibrium by the chi-square test. Unconditional multiple logistic regression models were used to calculate odds ratios (ORs) and 95 % confidence intervals (CIs) for the association of genotype with endometrial cancer risk. Genotype data were analyzed with the homozygote of the common allele as the reference group. Variants of homozygotes and heterozygotes were combined to evaluate the dominant effect. For each SNP, trend tests were conducted by assigning the ordinal values 1, 2, and 3 to homozygous wild-type, heterozygous, and homozygous variant genotypes, respectively, and by adding these scores as a continuous variable in logistic regression model. All multivariate models were adjusted for age, family history, obesity, smoking status, parity, menopausal status, and use of contraceptive and menopausal hormones. Since levels of TopBP1 mRNA and protein expression in studied material specimens did not show normal distribution (Kolmogorov-Smirnov test) the non-parametrical statistical tests (Mann–Whitney U test, Kruskal-Wallis test with *post hoc* multiple comparisons or the Spearman rank correlation test) were applied. The chi square test was used to identify relationship between positive expression of TopBP1 and clinicopathological parameters. Reported p-values were two-sided. Probabilities were considered significant whenever p-value was lower than 0.05. All analyses were completed using SAS software (version 9.0 SAS Institute, Cary, NC, USA).

## Results

### Genotypes and Genotypic Distribution in Patients and Control Subjects

Genotype distributions for TopBP1 polymorphisms in 121 endometrial cancer patients and 556 control subjects are summarized in Table 2. Three SNPs (rs185903567, rs116195487, and rs112843513) were not observed in the 3′UTR of *TopBP1* gene in our studied controls and cases. The rs116645643 was observed only in control group, but it was not included in the further analyzes. For the control group, rs116645643 polymorphism was in Hardy-Weinberg equilibrium (*p* = 0.86).

**Table 2:** Frequency distribution of the TopBP1 genotypes/alleles in cases and controls, and the risk of endometrial cancer

Variables	Cases (%)/Controls (%)	OR (95 % CI)^a^	p*
rs115160714			
C/C	112 (92.6)/548 (98.6)	1.00 (ref.)	0.0003
C/T	8 (6.6)/7 (1.2)	5.59 (1.96–15.91)	0.21
T/T	1 (0.8)/1 (0.2)	4.89 (0.30–79.23)	
C	232 (99.2)/1103 (99.2)	1.00 (ref.)	
T	10 (7.4)/9 (0.8)	5.28 (2.12–13.14)	0.0001
p- trend^b^		0.0003	
C/T or T/T vs. C/C^c^		5.50 (2.05–14.74)	0.0001
C/T or C/C vs. T/T^d^		4.62 (0.29–74.46)	0.28

^*^ *p* value for additive models

^a^Adjusted for age

^b^Testing additive genetic model (Cochran-Armitage test for trend)

^c^Testing dominant genetic model

^d^Testing recessive genetic model

All cases and controls were common allele carriers. Only one SNP (rs115160714) showed an association with endometrial cancer. The tested SNP did not follow the Hardy-Weinberg equilibrium for control group. The minor allele frequencies among the controls were consistent with those of the general population, as found in the NCBI dbSNP databases. The minor allele frequencies of rs115160714 were less than 5 %. The frequency of individuals who carried (T) allele was significantly higher in cases group (7.4 %) than in control group (0.8 %; *p* < 0.0001). Compared to homozygous common allele carriers, heterozygous for the T variant were found to be at a significant 5.59-fold increased risk of breast cancer (95 % CI = 1.96–15.91; *p* = 0.0003).

### Association of rs115160714 with Clinical and Environmental Parameters

Of the 121 endometrial cancer patients, 102 (84.3 %) had a low-grade tumor (grades G1 and G2), and 19 (15.7 %) had a high-grade tumor (G3). Most tumors, (96–79.3 %) were classified as FIGO I and II, and the remaining 25 (20.7 %) were FIGO III/IV (Table 3). There was a significant association between CT genotype and tumor grade or FIGO classification. Most carriers of minor allele had a high-grade tumors classified as FIGO III/IV (Table 3).

**Table 3:** Adjusted odds ratio for relation between TopBP1 genotypes and different tumor grades and FIGO classifications

Variables	Grade (n, %)		OR (95 % CI)^a^	p*
	Low grade (*n* = 102)	High grade (*n* = 19)		
CC	100 (98.0)	12 (63.1)	1.00 (ref.)	
CT	2 (2.0)	6 (31.5)	25.17 (3.68–170.43)	0.0001
TT	0 (0.0)	1 (5.4)	−	−
C	200 (99.5)	30 (78.9)	1.00 (ref.)	
T	4 (0.5)	8 (21.1).	13.24 (3.5–50.57)	0.0001
	FIGO classification (n, %)			
	FIGO I/II (*n* = 96)	FIGO III/IV (*n* = 25)		
CC	92 (95.8)	20 (80.0)	1.00 (ref.)	
CT	4 (4.2)	4 (16.0)	4.61 (1.05–20.71)	0.03
TT	0 (0.0)	1 (4.0)	−	−
C	188 (97.9)	44 (88.0)	1.00 (ref.)	
T	4 (2.1)	6 (12.0)	6.43 (1.75–23.57)	0.005

^*^ *p* value for additive models

^a^Adjusted for age

### Association Between TopBP1 Genotypes and mRNA/Protein Expression in Endometrial Cancer Tissue

**Fig. 1:**
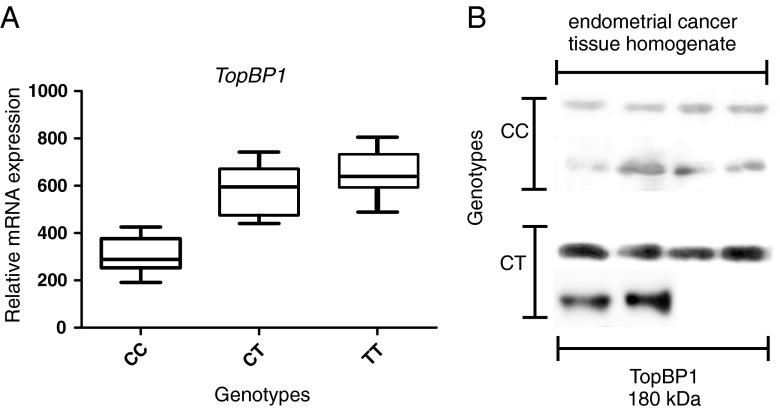
The relationship between TopBP1 mRNA and protein expression and the rs115160714 genotype in endometrial cancers. **a** Expression of *TopBP1* gene measured by real-time PCR in relation to genotype. **b** Western blotting analysis of TopBP1 expression measured in relation to genotype. Figure shows the representative results of TopBP1 immunodetection in endometrial cancer tissue homogenates (50 μg protein per lane)

We found that mean TopBP1 mRNA expression was lower in the case of individuals with the CC genotype than in case of minor allele carriers, i.e. CT heterozygotes and TT homozygotes (308.0 ± 117.0, 582.3 ± 142.0 and 673.6 ± 132.6 copies of TopBP1 mRNA per 1000 copies of GAPDH mRNA, respectively, *p* < 0.05 for all comparisons) (Table 4, Fig 1a). We found TopBP1 protein expression in 81.2 %, 75.0 % and 0.0 % of endometrial cancer tissue homogenate samples of CC, CT, and TT genotype carriers, respectively. Although the protein expression was more frequently observed in common allele carriers group, the mean expression level was lower than in minor allele carriers (122.4 ± 36.7, 215.1 ± 43.2, 189.3 ± 21.5 IOD relative units, respectively, *p* < 0.05 for all comparisons) (Table 4, Fig 1b). There was a statistically significant correlation between TopBP1 mRNA and protein expressions (Spearman correlation coefficient for CC and CT genotype 0.67 and 0.70, respectively, *p* < 0.05 for all comparisons). However, not in all cases with positive mRNA expression we could detected TopBP1 protein. Both mRNA and protein was detected in 73 of 112 CC samples, in 5 of 8 of CT samples and 0 of 5 TT samples.

**Table 4:** Comparison of TopBP1 mRNA and protein expression in endometrial cancer tissues with genotypes of *TopBP1* gene

Gene			
Genotypes	Positive expression (n, %)	*TopBP1* mRNA expression [copies of *TopBP1* mRNA per 1000 copies of *GAPDH* mRNA]	p^a^
CC	96/112 (85.7)	308.0 ± 117.0	
CT	7/8 (87.5)	582.3 ± 142.0	0.006
TT	1/1 (100.0)	673.6 ± 132.6	0.002
Protein			
	Positive expression (n, %)	TopBP1 protein expression [Integrated Optical Density (IOD) relative units] homogenate fraction	p^a^
CC	91/112 (81.2)	122.4 ± 36.7	
CT	6/8 (75.0)	215.1 ± 43.2	0.018
TT	0/1 (0.0)	189.3 ± 21.5	0.043

Results are given as mean ± standard error

^a^Differences between the three groups were evaluated with Kruskal-Wallis test with *post hoc* multiple comparisons

## Discussion

The DNA damage checkpoint is a signal transduction pathway that monitors the integrity of the DNA, prevents cell cycle progression and promotes appropriate DNA metabolism. Failure to trigger the DNA damage checkpoint leads to genome instability, one emerging hallmark of cancer. The ATM (ataxia telangiectasia mutated) and ATR (ATM and RAD3-related) kinases are two key DNA damage sensors. TopBP1 has been established as an essential activator of ATR. TopBP1 may regulate the activation of ATR-Chk1 checkpoint signaling at several levels i.e. TopBP1 is a direct activator of ATR kinase activity, TopBP1 acts as a scaffold for all necessary proteins for ATR activation, TopBP1 can regulate late stage of the DNA damage checkpoint by facilitating Chk1 phosphorylation and activated ATR [7].

There are only a few studies concerning the role of mutation in genes involved DNA damage checkpoint response in endometrial cancers. The results showed that *ATR* and *BRCA1* were frequently mutated in endometrial cancer patients [9]. Mutations in ATR were associated with biologic aggressiveness as evidenced by reduced disease-free and overall survival [10].

The biological functions of TopBP1 protein as well as its close similarity with BRCA1 prompted us to investigate whether alterations in TopBP1 gene can influence the risk of endometrial cancer. To our knowledge, there is no literature data on TopBP1 and endometrial cancer. Our earlier studies concerning TopBP1 in breast cancer showed association between SNP (rs115160714) with breast cancer risk as well as aberrant expression of this protein in breast cancer. Our findings suggested that increased level of TopBP1 protein might be associated with progression of hereditary breast cancer [11].

In this study, we demonstrated that out of five studied polymorphisms in the 3′UTR region of *TopBP1* gene only one, the rs115160714 was significantly associated with endometrial cancer risk. Compared to homozygous common allele carriers, heterozygous for the T variant were found to be at a highly significant 5.59 -fold increased risk of developing endometrial cancer (95 % CI = 1.96–15.91; *p* = 0.0003). This results are similar to that obtained earlier for breast cancer (OR = 3.54 (95 % CI = 1.56–8.39; *p* = 0.002) [8].

We found out that mean TopBP1 mRNA and protein levels were higher in case of individuals with CT genotype. We do not know the reason of this correlation. However, studied SNP (rs115160714) is located at the 3′UTR region TopBP1 gene which may cause higher production of mRNA. The other explanation is that this SNP change the half-life of mRNA leading to grater TopBP1 protein level. Since the rs115160714 is located in miRNAs (miR-3138, miR-4302 and miR-1207-5p) binding site, increased level of TopBP1 mRNA might possibly results from altered posttranscriptional regulation of gene expression.

The studies of Liu et al. [12] showed that TopBP1 plays a role in repressing of p53. TopBP1 interacts with p53 binding domain and inhibits the promoter binding activity of p53. Thus, increased expression of TopBP1 may cause deregulation of this important tumor suppressor protein and contribute to cancer development or progression.

In conclusion, our results showed that rs115160714 is associated with increased expression of TopBP1 and endometrial cancer risk.

## References

[1] Amant F, Moerman P, Neven P, Timmerman D, Van Limbergen E, Vergote I (2005). Endometrial cancer. Lancet.

[2] Segev Y, Iqbal J, Lubinski J (2013). Hereditary Breast Cancer Study Group. The incidence of endometrial cancer in women with BRCA1 and BRCA2 mutations: an international prospective cohort study. Gynecol Oncol.

[3] Forma E, Bryś M, Krajewska W, Kruman I (2011). TopBP1 in DNA damage response. DNA repair/book 4.

[4] Glover JNM (2006). Insights into the molecular basis of human hereditary breast cancer from studies of the BRCA1 BRCT domain. Fam Cancer.

[5] Sokka M, Parkkinen S, Pospiech H, Syväoja JE (2010). Function of TopBP1 in genome stability. Subcell Biochem.

[6] Rodriguez MC, Songyang Z (2008). BRCT domains: phosphopeptide binding and signaling modules. Front Biosci.

[7] Smits VA, Warmerdam DO, Martin Y, Freire R (2010). Mechanisms of ATR-mediated checkpoint signalling. Front Biosci.

[8] Forma E, Brzeziańska E, Krześlak A (2012). Association between the c.*229C>T polymorphism of the topoisomerase IIβ binding protein 1 (TopBP1) gene and breast cancer. Mol Biol Rep.

[9] Bilbao C, Ramírez R, Rodríguez G (2010). Double strand break repair components are frequent targets of microsatellite instability in endometrial cancer. Eur J Cancer.

[10] Zighelboim I, Schmidt AP, Gao F (2009). ATR mutation in endometrioid endometrial cancer is associated with poor clinical outcomes. J Clin Oncol.

[11] Forma E, Krześlak A, Bernaciak M, Romanowicz-Makowska H, Bryś M (2012). Expression of TopBP1 in hereditary breast cancer. Mol Biol Rep.

[12] Liu K, Bellam N, Lin HY (2009). Regulation of p53 by TopBP1: a potential mechanism for p53 inactivation in cancer. Mol Cell Biol.

